# Honokiol inhibits the growth of head and neck squamous cell carcinoma by targeting epidermal growth factor receptor

**DOI:** 10.18632/oncotarget.4178

**Published:** 2015-05-19

**Authors:** Tripti Singh, Nirzari A. Gupta, Su Xu, Ram Prasad, Sadanandan E. Velu, Santosh K. Katiyar

**Affiliations:** ^1^ Department of Dermatology, University of Alabama at Birmingham, Birmingham, AL, USA; ^2^ Department of Chemistry, University of Alabama at Birmingham, Birmingham, AL, USA; ^3^ Comprehensive Cancer Center, University of Alabama at Birmingham, Birmingham, AL, USA; ^4^ Birmingham Veterans Affairs Medical Center, Birmingham, AL, USA

**Keywords:** oncology, carcinogenesis, chemotherapy, targeted therapy, signal transduction, prevention, animal model

## Abstract

Here, we report the chemotherapeutic effect of honokiol, a phytochemical from *Magnolia* plant, on human head and neck squamous cell carcinoma (HNSCC). Treatment of HNSCC cell lines from different sub-sites, SCC-1 (oral cavity), SCC-5 (larynx), OSC-19 (tongue) and FaDu (pharynx) with honokiol inhibited their cell viability, which was associated with the: (i) induction of apoptosis, (ii) correction of dysregulatory cell cycle proteins of G0/G1 phase. Honokiol decreased the expression levels of epidermal growth factor receptor (EGFR), mTOR and their downstream signaling molecules. Treatment of FaDu and SCC-1 cell lines with rapamycin, an inhibitor of mTOR pathway, also reduced cell viability of HNSCC cells. Administration of honokiol by oral gavage (100 mg/kg body weight) significantly (*P* < 0.01-0.001) inhibited the growth of SCC-1 and FaDu xenografts in athymic nude mice, which was associated with: (i) inhibition of tumor cell proliferation, (ii) induction of apoptosis, (iii) reduced expressions of cyclins and Cdks, and (iv) inhibition of EGFR signaling pathway. Molecular docking analysis of honokiol in EGFR binding site indicated that the chemotherapeutic effect of honokiol against HNSCC is mediated through its firm binding with EGFR, which is better than that of gefitinib, a commonly used drug for HNSCC treatment.

## INTRODUCTION

Squamous cell carcinoma of head and neck (HNSCC) is a commonly occurring malignancy world-wide. Despite the advances in conventional therapies, the overall survival rate for HNSCC has only been modestly improved. Mortality rate has remained approximately 50% for the past three decades and is responsible for approximately 20,000 deaths in the United States annually [1-5]. Available therapies, including conventional chemotherapy and surgical resection, are often associated with considerable toxicity, drug resistance, impairment of speech and swallowing functions [6-9]. Therefore, the developments of new and promising strategies or complementary and alternative therapies are urgently needed. These new treatments can be given in combination with available treatment options to lower the doses of toxic drugs and to overcome drug resistance. An effective treatment strategy must ensure treatment efficacy, reduced toxicity and improved quality of life in the patients suffering from HNSCC. Chemoprevention of cancer can be considered as an appropriate complementary and alternative strategy for HNSCC treatment.

Epidermal growth factor receptor (EGFR) has been reported to be overexpressed in approximately 90% of HNSCC, and the overexpression of EGFR has been associated with poor clinical outcomes of HNSCC [10-12]. Therefore, EGFR is considered as a promising target for the treatment of patients suffering from HNSCC. Cetuximab (an FDA approved drug for the treatment of HNSCC) and some other investigational drugs which include the use of small molecule inhibitors (e.g., gefitinib) target EGFR. However, the poor response rates, toxicity and resistance of these drugs or inhibitors have limited their use as therapeutic agents for HNSCC [13, 14]. Therefore, development of less toxic and less resistance-associated alternative treatment options is urgently needed.

Some natural phytochemicals that are non-toxic in nature with medicinal values can be considered as promising candidates for the prevention and treatment of cancers. These phytochemicals can be used as complementary and alternative medicine and/or as adjuvant therapy for the conventional cytotoxic therapies. Honokiol (C_18_H_18_O_2_), a biphenolic compound, is such a phytochemical and is a bioactive constituent found in the bark and leaves of *Magnolia* plant. The diverse biological and pharmacological activities, such as anti-inflammatory, antifungal, anti-oxidative and anti-carcinogenic, of honokiol have been investigated in recent years [15-19]. The chemotherapeutic and chemopreventive effects of honokiol have been reported previously in several tumor models, including skin, breast, melanoma, non-small cell lung cancer and prostate [15-19]. Anti-carcinogenic effect of honokiol was also determined against HNSCC cells using *in vitro* and *in vivo* models and EGFR was recognized as a molecular target [13]. However, the anti-carcinogenic potential of honokiol with definitive EGFR binding using molecular docking analysis and molecular mechanism has not been explored in HNSCC. We hypothesize that honokiol inhibits the growth of HNSCC cells by targeting and binding firmly with EGFR. To test our hypothesis, we assessed the chemotherapeutic effect of honokiol on HNSCC cell lines derived from different sub-sites, such as larynx (UM-SCC5), pharynx (FaDu), tongue (OSC19) and oral cavity (UM-SCC1) [20].

## RESULTS

### Honokiol inhibits cell viability of HNSCC cells

**Figure 1:**
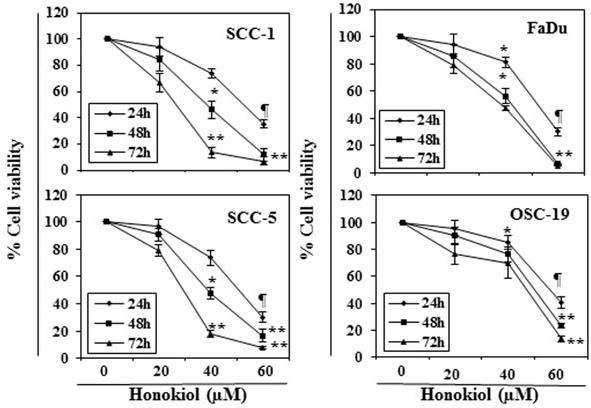
*In vitro* treatment of HNSCC cells with honokiol inhibits the cell viability in a dose- and time-dependent manner The HNSCC cells (SCC-1, SCC-5, OSC-19 and FaDu) were treated with various concentrations of honokiol (0, 20, 40 and 60 μM) for 24 h, 48 h and 72 h and cell viability was determined using the MTT assay. The data are expressed in terms of percent of control cells (non-honokiol-treated) as the mean ± SD of 5 replicates. Significant difference *vs.* control group, *P < 0.05; ^¶^*P* < 0.01; ^**^P < 0.001.

The effect of honokiol on viability of HNSCC cells, SCC-1, SCC-5, OSC-19 and FaDu, were determined using an MTT assay. The cells were treated with different concentrations of honokiol (0, 20, 40 and 60 μM) for 24, 48 and 72 h. A dose- and time-dependent inhibition in viability of HNSCC cells was observed, as shown in Figure 1. The reduction in the viability of the SCC-1 and FaDu cells observed after treatment with honokiol ranged respectively from 16% to 89% (*P* < 0.001) and 15% to 94% (*P* < 0.001) after 72 h (Figure 1). Under identical conditions, similar effects were observed on treatment of SCC-5 and OSC-19 cells with honokiol.

### Treatment of HNSCC cells with honokiol induces apoptosis

**Figure 2:**
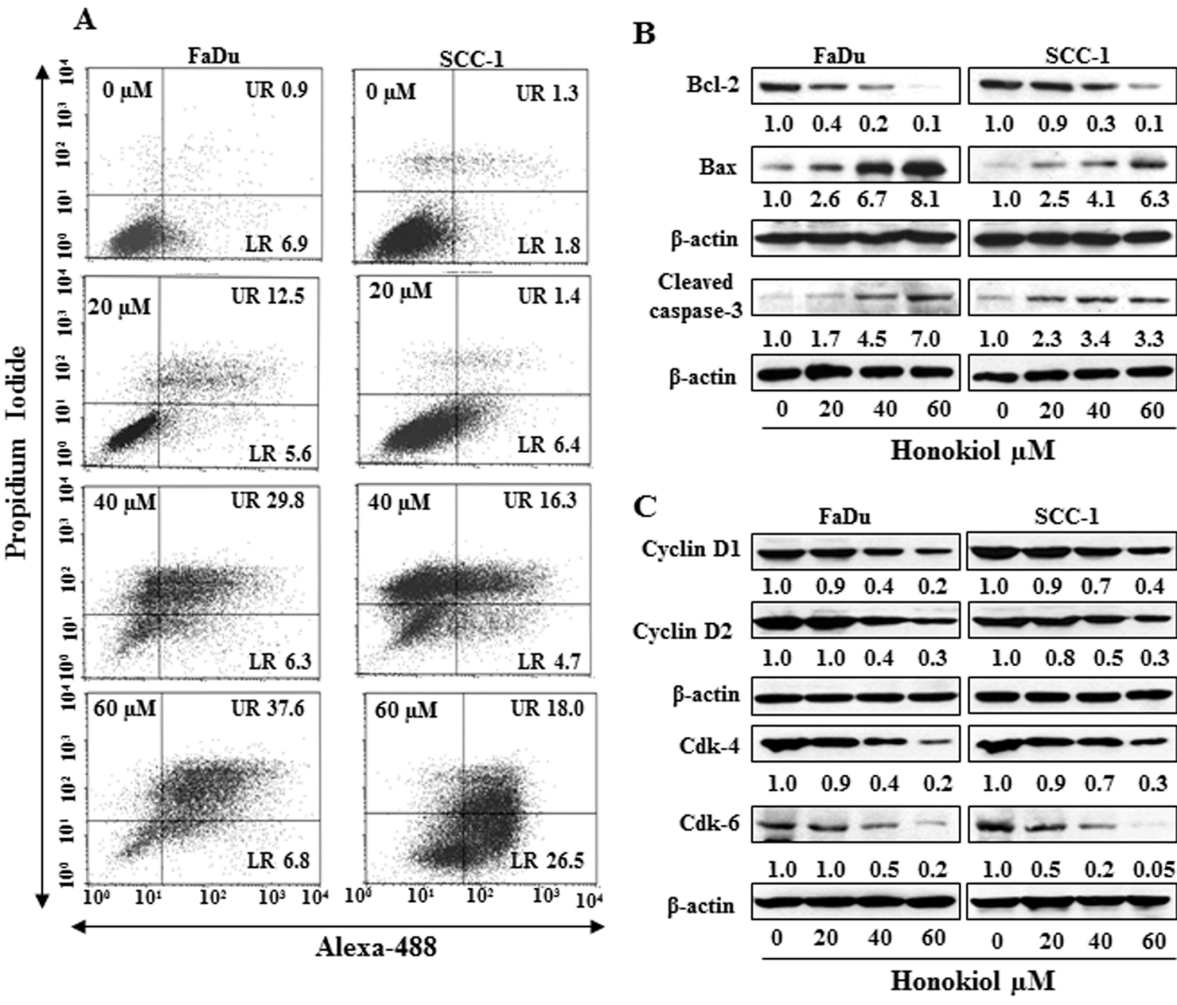
A Treatment of FaDu and SCC-1 cells with different concentrations of honokiol for 48 h induces apoptosis. Apoptosis was determined using Annexin V-Alexa Fluor488 (Alexa488) Apoptosis Vybrant Assay Kit. Lower right (LR) quadrant indicates the percentage of early apoptotic cells, while Upper right (UR) quadrant indicates the percentage of late apoptotic cells. **B.** *In vitro* treatment of FaDu and SCC-1 cells with honokiol for 48 h resulted in a dose-dependent effect on the proteins of Bcl-2 family and cleaved caspase-3, as determined using western blot analysis. **C.**, Effect of honokiol on the proteins of G0/G1 cell cycle phase after the treatment of cells for 48 h.

FACS analysis was performed to quantitate the percentage of apoptosis in HNSCC cells. As honokiol induced inhibition of cell viability was almost similar in all the four cell lines studied, FaDu and SCC-1 cell lines were selected for further investigation. FaDu and SCC-1 cell lines were treated with various doses of honokiol and quantitative analysis of apoptosis was determined using the Alexa488 Apoptotic Cell Detection Kit using flow cytometry, as detailed previously [20]. The number of cells undergoing apoptosis was determined in terms of the percentage of early-stage and late-stage apoptotic cells, which are shown in lower right (LR) and upper right (UR) quadrants of the FACS histogram, respectively (Figure 2A), and as detailed previously [21]. Treatment of the FaDu and SCC-1 cells with honokiol for 48 h resulted in a significant induction of apoptotic cell death in both cell lines. The percentages of total apoptotic cells (UR+LR quadrants) in FaDu cells after honokiol treatment ranged from 18.1% (20 μM) to 44.4% (60 μM) compared to only 7.8% in non-honokiol-treated control cells. Similar range of apoptotic cell death after honokiol treatment was observed in SCC-1 cells (Figure 2A).

Cancer cell apoptosis is tightly regulated by functions of the proteins of Bcl-2 family, and proteins of Bcl-2 family act as promoters or inhibitors of cell death [22-24]. Western blot analysis and subsequently measurement of band densities revealed that treatment of FaDu and SCC-1 cells with honokiol (0, 20, 40, 60 μM) for 48 h resulted in a dose-dependent decrease in the expression of anti-apoptotic protein Bcl-2 whereas the expression of pro-apoptotic protein Bax was increased with increasing doses of honokiol treatment (Figure 2B). As shown in Figure 2B, honokiol treatment also resulted in an increase in activated form of caspase-3 in both FaDu and SCC-1 cell lines.

### Honokiol decreases the expression levels of cyclins and Cdks in HNSCC cells

Cyclins and Cdks have been implicated to play important roles in cell cycle regulation and that affect the proliferation of cells [25, 26]. Therefore, the effect of honokiol was determined on the levels of cyclins and Cdks of G0/G1 phase following the treatment of FaDu and SCC-1 cells with honokiol for 48 h. Western blot analysis revealed that treatment of these cells with honokiol (0, 20, 40 and 60 μM) resulted in a concentration-dependent decrease in the expression levels of cyclin D1 and cyclin D2. Similarly, decrease in the levels of Cdk4 and Cdk6 proteins was also observed after honokiol treatment, as shown in Figure 2C.

### Honokiol decreases the levels of EGFR, p-EGFR and downstream targets of EGFR signaling pathway in HNSCC cells

**Figure 3:**
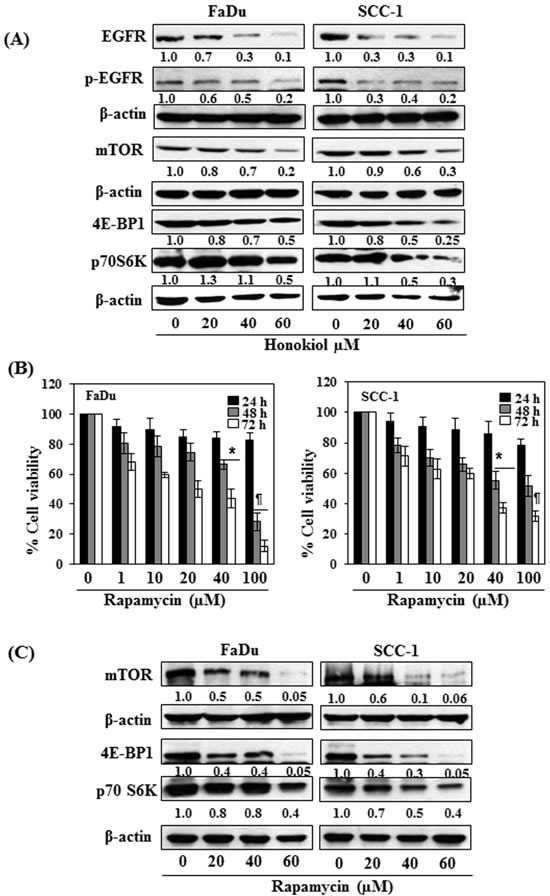
A FaDu and SCC-1 cells were treated with various concentrations of honokiol for 48 h. Cell lysates were prepared and subjected to western blot analysis for the analysis of different proteins of EGFR and mTOR signaling pathways. **B.** FaDu and SCC-1 cells were treated with different concentrations of Rapamycin (an inhibitor of mTOR) for 24 h, 48 h and 72 h and cell viability was determined using MTT assay. Rapamycin inhibits cell viability in HNSCC cells in a dose- and time-dependent manner. The data on cell viability are expressed in terms of percent of non-rapamycin-treated control cells as the mean± SD of 4 replicates. Significant difference *vs.* control group, *P < 0.05; ^¶^*P* < 0.001. **C.** Effect of rapamycin on the levels of different proteins of mTOR signaling pathway in FaDu and SCC-1 cells after treatment for 48 h.

As it has been shown that EGFR is overexpressed in approximately 90% of HNSCC [10-12], the effect of honokiol was determined on EGFR in HNSCC cells after treating the FaDu and SCC-1 cells with honokiol for 48 h. Western blot analysis and measurement of band densities indicated that treatment of FaDu cells with honokiol resulted in reduction in the levels of EGFR and p-EGFR in a dose-dependent manner (Figure 3A). Similar effect of honokiol on the levels of EGFR and p-EGFR was also observed when SCC-1 cells were treated with honokiol (Figure 3A).

As mTOR and its downstream signaling molecules are the downstream targets of EGFR signaling mechanism, we further checked whether inhibition of EGFR by honokiol also affect the expression levels of mTOR and associated signaling proteins. As shown in Figure 3A, treatment of both FaDu and SCC-1 cells with different concentrations of honokiol (0, 20, 40 and 60 μM) decreased the levels of mTOR as well as the protein levels of 4E-BP1 and p70S6K in both cell lines in a dose-dependent manner. The pattern of inhibitory effect of honokiol on these proteins was identical in both cell lines.

### Rapamycin, an inhibitor of mTOR signaling, inhibits cell viability of FaDu and SCC-1 cells

To verify honokiol-mediated adverse effects on cell viability in HNSCC cells through EGFR/mTOR pathway, FaDu and SCC-1 cells were treated with rapamycin (0, 1, 10, 20, 40 and 100 μM) for 24, 48 and 72 h, and the effect on cell viability was determined using MTT assay. Treatment of cells with rapamycin had no significant effect on cell viability after the treatment of cells for 24 h, while rapamycin reduced the cell viability in FaDu (20%-72%) and SCC-1 (22%-49%) cells after 48 h treatment. The inhibitory effect of rapamycin on cell viability was greater at 72 h time point than at 48 h (Figure 3B). The effect of rapamycin was also determined on the mTOR and its down-stream protein targets after treating the cells with rapamycin for 48 h. The protein expression levels of mTOR, 4E-BP1 and p70SK6 were decreased in a dose-dependent manner after treatment with rapamycin as analyzed by western blotting (Figure 3C), suggested the involvement of mTOR pathway in inhibition of cell viability in SCC-1 and FaDu cells by rapamycin.

### siRNA knockdown of EGFR results in suppression of HNSCC cell viability

**Figure 4:**
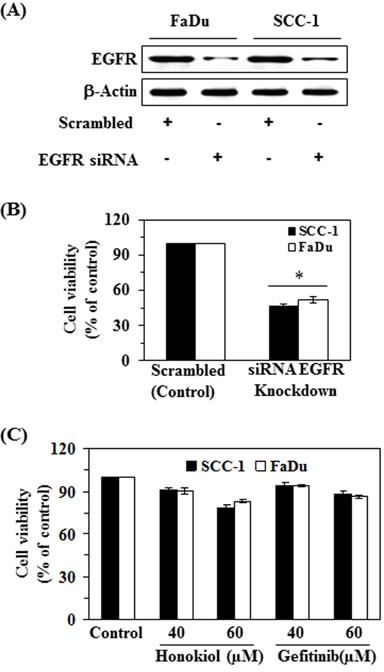
siRNA knockdown of EGFR from FaDu and SCC-1 cells leads to a reduction in cell viability **A.** FaDu and SCC-1 cell lines were transfected with EGFR siRNA. The levels of EGFR in the lysates of both cell lines were checked using western blot analysis. **B.** Cell viability was determined in EGFR siRNA transfected cells using MTT assay, and is presented in terms of percent of control (scrambled). Significant suppression *versus* control, *P < 0.01. **C.** EGFR siRNA transfected cells were subjected to the analysis of cell viability using MTT assay after treatment with and without honokiol and gefitinib for 48 h.

In order to better understand the role of EGFR in honokiol-mediated suppression of HNSCC cell viability, the FaDu and SCC-1 cell lines were subjected to knockdown of EGFR using siRNA transfection kit following the manufacturer's instructions. The downregulation of EGFR in cell lines was verified using western blot analysis (Figure 4A). Transfected and control cells were grown and cell viability was determined in both cell lines using MTT assay. MTT assay analysis indicated that the cell viability or proliferation potential of EGFR knocked down cells was significantly decreased (*P* < 0.01) compared to control cells, as shown in Figure 4B. Further, the treatment of EGFR siRNA transfected SCC-1 and FaDu cells with honokiol or gefitinib (an inhibitor of EGFR) for 48 h did not result in significant inhibition of cell viability compared to cells not treated with honokiol or gefitinib (control cells) (Figure 4C).

### Administration of honokiol by oral gavage inhibits the growth of HNSCC tumor xenografts: honokiol is non-toxic to mice

**Figure 5:**
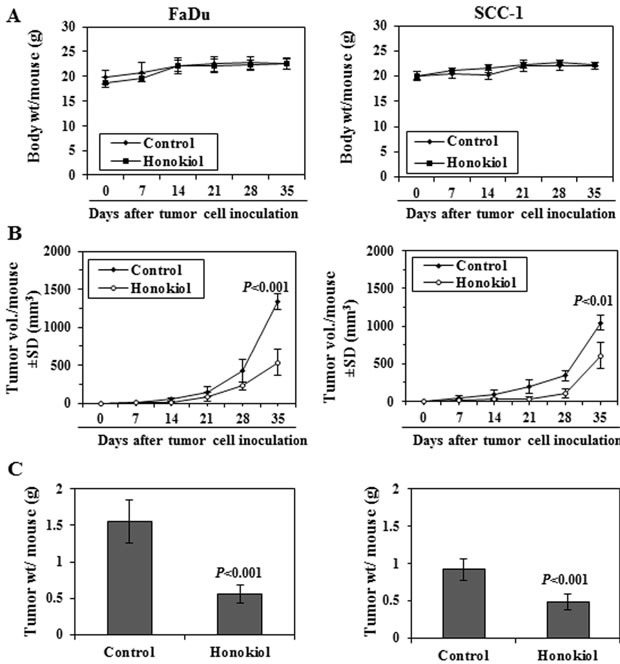
Administration of honokiol by oral gavage inhibits the growth of FaDu and SCC-1 cells grown as tumor xenograft in athymic nude mice **A.** Body weight of mice in different treatment groups were recorded on weekly basis. The body weight of mice in control (vehicle-treated) and honokiol (100 mg/kg body weight) treated groups did not vary significantly throughout the duration of *in vivo* tumor xenograft experiment. **B.** Size of the tumor xenografts were recorded on weekly basis in each treatment group. Average tumor volume (mm^3^) ± SD (*n* = 6) was calculated from each group and was plotted as a function of time. Significant inhibition in tumor xenograft growth was recorded in the honokiol treated mice compared with the mice treated with vehicle alone. Significant difference vs control group at the termination of the experiment, *P* < 0.01 or *P* < 0.001. **C.** At the termination of the experiment, total tumor xenograft tissue was harvested from each mouse and the wet tumor weight was measured on a digital balance. Tumor weight is reported as the mean ± SD (g), *n* = 6. Significant inhibition in tumor weight in honokiol treated mice *vs* vehicle-treated control mice, *P <* 0.001.

Next, we determined the effect of honokiol on FaDu and SCC-1 xenografts growth using athymic nude mice. Honokiol was administered by oral gavage (100 mg/kg body weight of mice) and the effects on the growth of the tumor xenografts were monitored. The daily consumption of drinking water and AIN76A diet by the mice in each group was identical (data not shown) and the mice that were administered honokiol did not exhibit impaired movement and/or any other visible sign of physical toxicity. The average body weight of the honokiol-administered and non-honokiol-administered control mice remained almost identical throughout the 5 weeks duration of the experimental protocol (Figure 5A).

The treatment of mice with honokiol resulted in reduced growth of both FaDu and SCC-1 xenografts. As shown in Figure 5B, administration of honokiol significantly inhibited the growth of FaDu tumor xenografts in the nude mice (59%, *P* < 0.001, *n* = 6) compared with vehicle-treated control mice. Administration of honokiol at the same dose also resulted in a significant inhibition (42%, *P* < 0.01) in the growth of SCC-1 tumor xenograft (Figure 5B, right panel). At the termination of the experiment on the 35^th^ day of the experiment, the mice were sacrificed, tumors were harvested and the wet weight of the tumor/mouse in each treatment group was weighed on digital balance. The wet weight of the FaDu tumors was 64% lower (*P* < 0.001) in mice administered honokiol compared with non-honokiol-treated control mice (Figure 5C, left panel). Administration of honokiol at the same dose also resulted in a significant reduction (42%, *P* < 0.001) in the wet weight of the SCC-1 tumor xenografts (Figure 5C, right panel) compared to non-honokiol-treated control mice.

### The inhibition of tumor xenografts growth by honokiol was associated with its inhibitory effects on PCNA and alterations in proteins of Bcl-2 family

**Figure 6:**
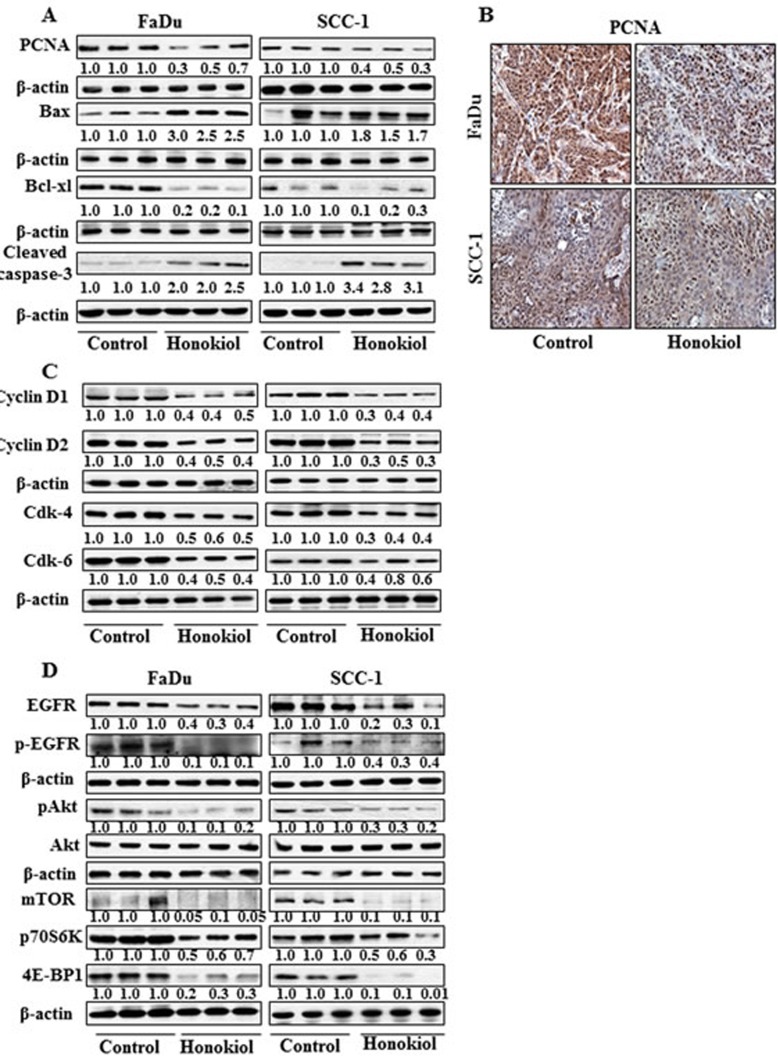
At the termination of tumor xenograft experiment, tumor tissues were harvested and tumor lysates were prepared for the analysis of various proteins associated with apoptosis, cell cycle regulatory proteins, EGFR and mTOR signaling pathways using western blot analysis Resultant data are presented in triplicates where each tumor lysate sample was prepared by pooling the tumor tissues from two mice. **A.** treatment of mice with honokiol by oral gavage inhibits the levels of PCNA, and Bcl-xl while increased the levels of pro-apoptotic protein Bax in tumors. **B.** Immunohistochemical detection of PCNA-positive cells in tumor tissues from FaDu and SCC-1 tumor xenografts. **C.** Effect of honokiol treatment on G0/G1 cell cycle regulatory proteins, cyclins and Cdks, in tumor tissues and compared with vehicle-treated controls. **D.** Treatment of mice with honokiol inhibits the levels of EGFR, pAkt, and proteins of mTOR signaling pathway in tumor xenograft tissues compared to the tumor samples from vehicle-treated control group of mice.

Western blot analysis and subsequent measurements of band densities in different blots of tumor lysates indicate that treatment of mice with honokiol inhibited the levels of PCNA in tumors compared with the tumor samples from non-honokiol-treated control mice (Figure 6A). These observations were further verified by immunohistochemical detection of PCNA-positive cells in tumor xenograft samples from FaDu and SCC-1 cells. As shown in Figure 6B, the intensity of PCNA-positive cells was lower in the tumor samples from honokiol-treated animals compared to the tumors from control animals. Another reason of reduced growth of tumor xenografts by honokiol treatment may be due to the apoptotic cell death of cancer cells. As shown in Figure 6A, the levels of the anti-apoptotic protein, Bcl-xl, was reduced while the levels of the pro-apoptotic protein, Bax, were higher in xenograft tumors from the animals treated with honokiol than tumors from control mice. Additionally, the levels of activated caspase-3 were higher in the tumor samples from the mice treated with honokiol compared to tumor samples from control mice.

### Administration of honokiol reduces the expressions of cell cycle regulatory proteins of G0/G1 phase in tumor xenograft tissues

As the cell cycle regulatory proteins of G0/G1 phase play a critical role in tumor cell growth [25, 26], we determined the effect of honokiol on the regulatory proteins of G0/G1 phase in tumor xenograft samples. As shown in Figure 6C, treatment of mice with honokiol resulted in a marked decrease in the expressions of cyclin D1 and cyclin D2 and reduced expressions of Cdk4 and Cdk6 in tumor samples obtained from honokiol-treated mice compared to tumor samples from control mice.

### Honokiol modulates the expression levels of EGFR and mTOR signaling pathways in tumor xenograft of HNSCC cells

Western blot analysis revealed that treatment of mice with honokiol inhibited the levels of EGFR as well as p-EGFR in tumor xenograft samples from both FaDu and SCC-1 cells compared to the tumor samples from the control mice (Figure 6D). Honokiol treatment also decreased the levels of pAkt in tumors while the levels of Akt remained unchanged. Further, inhibitory effect of honokiol on EGFR in tumor xenografts was also associated with reduced expressions of mTOR and its down-stream target proteins p70S6K and 4E-BP1, suggesting the involvement of these molecular targets in preventing the *in vivo* growth of HNSCC xenografts by honokiol in mice (Figure 6D).

### Molecular docking analysis: Binding of honokiol in the EGFR binding site

**Figure 7:**
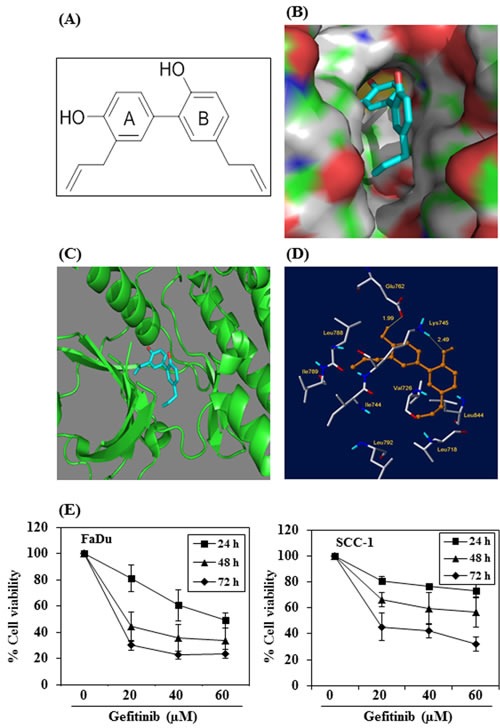
Molecular docking analysis of honokiol with EGFR **A.** Chemical structure of honokiol. **B.** The surface-docking model of honokiol in the EGFR active site. **C.** Ribbon representation (3D) of the protein structure along with binding of honokiol. **D.** The critical interaction of honokiol in the EGFR active sites as predicted by Surflex-Dock model. **E.** The effect of gefitinib on viability of FaDu and SCC-1 cells. Treatment of cells with varying concentrations of gefitinib inhibits the viability of cells in a dose- and time-dependent manner.

We determined whether the anti-HNSCC effect of honokiol is due to the binding between honokiol (Figure 7A, molecular structure) and EGFR molecules. For this purpose we used molecular docking analysis as detailed in Materials and methods. The docking model of honokiol in EGFR showed that it fits snuggly in the binding site as shown in the Figure 7B. The reference ligand gefitinib was also docked in the active site of EGFR for comparison purposes (data not shown). Honokiol showed better affinity with EGFR compared to gefitinib as indicated by the binding scores obtained from Surflex docking. Higher binding scores show better binding of the compound with the protein. Honokiol has a binding score of 5.5471 as compared to 4.3014 for gefitinib. Critical interactions of honokiol in the EGFR binding site were also examined (Figure 7C). The OH groups of honokiol were found to have two important hydrogen bond interactions within the active site. The first hydrogen bond was between OH group of ring A of honokiol (Figure 7A) with Glu762 (1.99 Å) and the second hydrogen bond was between the OH group of ring B and Lys745 (2.49 Å) (Figure 7D). In addition to this, the two hydrophobic chains on the rings A and B of honokiol were found to occupy two hydrophobic pockets in the binding site. The chain on ring A of honokiol interacted with hydrophobic residues such as Leu788, Ile789 and Ile744 and the second hydrophobic chain on ring B of honokiol interacted with hydrophobic residues such as Leu718, Val726, Leu844 and Leu792.

Further, as we found that honokiol has better binding affinity with EGFR than gefitinib, we determined the effect of gefitinib on the cell viability of FaDu and SCC-1 cells and compared with honokiol using identical experimental conditions as were used with honokiol (Figure 1). For this purpose, FaDu and SCC-1 cell lines were treated with gefitinib (0, 20, 40 and 60 μM) for 24, 48 and 72 h, and cell viability was determined using MTT assay. MTT assay results indicated that treatment of FaDu and SCC-1 cells with gefitinib resulted in inhibition of cell viability in a dose- and time-dependent manner (Figure 7E). However, the inhibitory effect of gefitinib on HNSCC cell viability was lower than that of honokiol at equimolar concentrations (Figure 1).

## DISCUSSION

HNSCC is a challenging clinical problem due to the persisting high rate of this malignancy. To develop newer and more effective candidates or drugs for HNSCC, we examined the effects of honokiol on various cell lines of HNSCC using both *in vitro* and *in vivo* models. Here, we report that honokiol significantly decreased the viability and induced apoptosis in various cell lines of human HNSCC, which were derived from tongue, larynx, oral cavity and pharynx, suggesting that honokiol possesses broad therapeutic effect on this malignancy. However, we did not find significant inhibition of cell proliferation of normal human bronchial epithelial cells (BEAS-2B) after honokiol treatment [18]. A major apoptotic signal transduction cascade associated with induction of apoptosis includes the proteins of Bcl-2 family, which either promote cell survival or promote apoptotic cell death [27, 28]. We found that induction of apoptosis in FaDu and SCC-1 cells by honokiol was associated with a decrease in the levels of anti-apoptotic proteins and a simultaneous increase in the pro-apoptotic proteins. Suppression of anti-apoptotic proteins of this family preserves the integrity of the mitochondria. Control of cell cycle progression in cancer cells is considered to be an effective strategy for the control of cancer growth [25, 26]. Our *in vitro* data indicated that treatment of HNSCC cells with honokiol affects the cell cycle proteins of G0-G1 phase, which indicates that one of the mechanisms by which honokiol may act to inhibit the cell viability or proliferation of HNSCC cells is down-regulation of the cell cycle regulatory proteins. Our finding of a marked decrease in the levels of cyclin D1, D2 and Cdks in FaDu and SCC-1 cells on honokiol treatment suggests the disruption of the uncontrolled cell cycle progression of these HNSCC cells. This act of honokiol may be one of the possible mechanisms of anti-carcinogenic effect in HNSCC cells. The increased expression of G1 phase cyclins in cancer cells provides an uncontrolled growth advantage because most of these cells either lack cyclin dependent kinase inhibitors (Cdki) or the expression of Cdki is not at a sufficient level to control Cdk-cyclin activity [29]. Apoptosis plays a crucial role in eliminating the hyper-proliferating neoplastic cells or mutated neoplastic cells from the system, and therefore is considered as a protective mechanism against cancer progression [30]. Therefore, honokiol seems to be a potent chemotherapeutic agent for HNSCC.

It is known that EGFR is over-expressed in majority of the HNSCC and its abolition is considered as a therapeutic effect of any drug in head and neck cancer clinically. Our study demonstrates that treatment of FaDu and SCC-1 cell lines with honokiol decreases the expression levels of total EGFR as well as p-EGFR and its downstream target mTOR. Activation of mTOR has been shown to contribute in tumor growth and progression. Thus, it can be suggested that inhibition of cell proliferation by honokiol is mediated, at least in part, through the downregulation of EGFR/mTOR signaling pathway. To further verify that inhibition of mTOR and its downstream targets by honokiol play a critical role in HNSCC growth, FaDu and SCC-1 cells were treated with rapamycin, an inhibitor of mTOR, and its effect on cell viability was examined. It was observed that treatment of cells with rapamycin results in significant inhibition of HNSCC cell viability as well as decrease in the levels of mTOR and its downstream targets. Additionally, treatment of EGFR siRNA transfected human HNSCC cells (FaDu and SCC-1) with honokiol or gefitinib did not result in significant inhibition of cell viability. Based on these observations, it appears that honokiol acts as an antagonist and/or causes increased turnover of EGFR, thus accounting for decreased expression of EGFR in HNSCC cells. These observations verify and support the evidence that honokiol inhibits the growth of HNSCC cells *in vitro* by targeting EGFR and its downstream molecular targets.

The therapeutic effect of honokiol against HNSCC was further examined and verified using *in vivo* pre-clinical model. These *in vivo* animal studies were necessary for the potential consideration of the use of honokiol in humans. Therefore an *in vivo* model of tumor xenografts was used to verify the chemotherapeutic potential of honokiol against HNSCC cell growth. The outcome of this study provides evidence that administration of honokiol by oral gavage inhibits the growth of FaDu and SCC-1 tumor xenografts without any apparent visible sign of toxicity in mice. Other investigators also have assessed the therapeutic effect of honokiol using tumor xenograft model but that study indicated that treatment of honokiol did not show significant inhibition of tumor xenograft growth in athymic nude mice [13]. In this study the investigators used only one HNSCC cell line, 1483, and it seems that the effect of honokiol was cell line-specific. Additionally, the *in vivo* dose of honokiol used in that study was lower than the dose of honokiol used by us in the current study. The identification of molecular targets is an important subject of consideration in terms of monitoring the clinical efficacy of cancer therapeutic agents and strategies in suggesting potential combinations with other therapeutic drugs. The inhibitory effect of honokiol on tumor xenograft growth was associated with the: (i) inhibition of PCNA, and correction of dysregulated proteins of cell cycle regulation, (ii) induction of apoptotic cell death of tumor cells, as indicated by the increased levels of activated caspase-3, and (iii) reduction in the levels of EGFR, pAkt and mTOR, including downstream targets of mTOR signaling in tumor xenograft samples collected at the termination of the *in vivo* animal experiments. Most importantly, (iv) we found and verified using molecular docking analysis that honokiol binds to EGFR firmly and this binding affinity of honokiol with EGFR was even greater than gefitinib, a commonly used drug for HNSCC. Conclusively, honokiol appears to be an attractive bioactive small molecule phytochemical for the management of head and neck cancer which can be used either alone or in combination with other available therapeutic drugs. However, more mechanism-based studies are required *in vivo* models with honokiol to further identify and verify the molecular targets associated with this malignancy.

## MATERIALS AND METHODS

### HNSCC cell lines and cell culture conditions

The HNSCC cell lines derived from the tongue (OSC-19), pharynx (FaDu), oral cavity (UM-SCC1) and larynx (UM-SCC-5) were obtained from Dr. Rosenthal (Department of Otolaryngology, University of Alabama at Birmingham, Birmingham, AL). No further authentication was done by the authors. Cells were grown as monolayer in Dulbecco's modified Eagle's medium supplemented with 10% FBS and 1% penicillin and streptomycin. For the treatment of cells, honokiol was dissolved in a small amount of ethanol (100 μl), diluted as required in culture medium (maximum concentration 0.1%, v/v) and added to sub-confluent cells.

### Honokiol, chemicals and antibodies

Purified honokiol was purchased from Quality Phytochemicals LLC (Edison, NJ). Antibodies specific for Bax, Bcl-2, Bcl-xl, cleaved caspase 3, cyclin D1, cyclin D2, mTOR, 4E-BP1, p70S6 kinase, pEGFR and EGFR were obtained from Cell Signaling Technology (Beverly, MA). Gefitinib and antibodies specific for PCNA, cyclin-dependent kinases (Cdk), β-actin and horseradish peroxidase conjugated rabbit anti-goat and goat anti-rabbit secondary antibodies were purchased from Santa Cruz Biotech (Santa Cruz, CA). The Annexin V-conjugated AlexaFluor488 Apoptosis Detection Kit was purchased from Molecular Probes, Inc. (Eugene, OR). The protein assay kit was procured from Bio-Rad (Hercules, CA). 3-[4,5-dimethyl-2-yl]-2,5-diphenyl tetrazolium bromide (MTT), rapamycin and all other chemicals were of analytical grade and obtained from Sigma Chemical Co. (St. Louis, MO).

### MTT assay for cell viability

The effect of honokiol on the viability of HNSCC cells was determined using MTT assay, as detailed previously [18, 20]. Gefitinib was dissolved in a small amount of dimethylsulfoxide (100 μl) then mixed in culture medium to achieve desired concentrations. The effect of honokiol and gefitinib on cell viability was calculated in terms of percent of control cells (non-honokiol- or non-gefitinib-treated), which was arbitrarily assigned a value of 100% viability.

### Apoptotic cell death analysis using flow cytometry

Honokiol- induced apoptotic cell death of HNSCC cells was determined quantitatively by flow cytometry using the Annexin V-conjugated Alexa fluor488 (Alexa488) Apoptosis Detection Kit following the manufacturer's protocol, also as described by us previously [20]. Briefly, cells were treated with different concentrations of honokiol for 48 h, then harvested, washed with PBS buffer and incubated with Alexa488 and propidium iodide. The stained cells were then analyzed by fluorescence activated cell sorting (FACS) using the FACSCalibur instrument (BD Biosciences).

### Western blot analysis and analysis of band density

Following treatment of HNSCC cells with or without honokiol the cells were harvested, and cell lysates were prepared [18, 20]. Similar to cell lysates, tumor xenograft tissues were homogenized in lysis buffer. Lysates thus prepared were subjected to western blot analysis to detect the levels of various proteins of interest, as described previously [18, 20]. Equal protein loading on the gel was verified using anti-β-actin antibody on the same membrane. In some cases, western blot membranes were cut into two or three parts according to the molecular weight of the proteins and subjected to incubation with the different antibodies, etc. Protein ladders were used as a molecular weight marker to identify different proteins. The relative density of each band in an immune-blot was measured using the ImageJ software (National Institute of Health). The numerical value of band density is shown under each blot, and the band density of control group was arbitrarily selected as ‘1’ and comparison was then made with densitometry values of other treatment groups.

### Transient transfection of EGFR siRNA in HNSCC cells

For functional analysis, the expression of EGFR in FaDu and SCC-1 HNSCC cells was knocked down using human-specific EGFR siRNA Transfection Reagent Kit (Santa Cruz Biotechnology, Santa Cruz, CA) following the manufacturer's protocol. Briefly, 2×10^5^ cells per well were seeded onto 6-well culture plates. The EGFR siRNA mix with transfection reagents or scrambled siRNA reagents were overlaid on the cells for seven h at 37°C and then transferred into 2x growth medium for about 24 h. After 24 h of transfection, cells were kept in a culture medium containing 2% FBS up to 48 h. The cells were then harvested and used for cell viability assays. Cells treated with scrambled probe were used as controls for the transfected cells. The knock down of EGFR expression in cells after transfection was confirmed using western blot analysis.

### Tumor xenograft study using athymic nude mice

Four-to-five week old (16-18 g/mouse) female immune-deficient athymic nude mice were purchased from the National Cancer Institute (NCI, Frederick, MD) and housed in the Animal Resource Facility at the University of Alabama at Birmingham in accordance with the Institutional Animal Care and Use Committee (IACUC) guidelines. Animal protocol was approved by the IACUC. To determine the *in vivo* chemotherapeutic efficacy of honokiol against HNSCC tumor xenograft growth, exponentially growing SCC-1 and FaDu cells (2×10^6^ in 100 μl PBS/mouse) were injected subcutaneously in the right flank of each mouse. One day after tumor cell inoculation, animals were divided randomly into two groups per cell line with six mice per group. Mice in Group I were treated with 100 mg honokiol/kg body weight of mouse in 200 μl of 0.5% carboxymethylcellulose (w/v) in 0.025% Tween-20 (v/v) solution in sterile water by oral gavage twice per week. This dose of honokiol for oral gavage was selected from the previous study where treatment of nude mice with honokiol significantly inhibited tumor xenograft growth of non-small cell lung cancer cells [18]. Mice in Group II received same vehicle only and served as vehicle treated control group. Mice were monitored for their body weight, diet and water consumption on regular basis throughout the experiment period. Tumor size was monitored twice per week. At the termination of experiment, mice were euthanized and tumors were harvested. Tumor size was measured by vernier calipers and volumes were calculated using the ellipsoid model formula: tumor volume = ½ (4π/3) (l/2) (w/2) h, where l = length, w = width and h = height. The wet weight of the tumor mass was recorded at the termination of the experiment using digital balance. One part of the tumor was used to prepare paraffin block for immunohistochemical analyses and other part was used to prepare tumor lysates for western blot analyses. Tumor samples from two mice were pooled and lysates were prepared in each experimental group, *n* = 6.

### Molecular docking analysis of honokiol

Molecular docking is a computational approach used to evaluate the interactions between ligand and its target. EGFR was identified as a possible target of honokiol as it decreased the expression levels of EGFR, p-EGFR and downstream targets of EGFR signaling pathway in HNSCC cells. In order to support this hypothesis, we carried out the docking analysis of honokiol with EGFR using the Surflex-Dock module of Sybyl X 1.2 software from Tripos Inc. (St. Louis, MO) [31]. Honokiol structure was built by using ‘sketch’ option in Sybyl X 1.2 and energy-minimized using conjugate gradient algorithm with a gradient convergence value of 0.01 kcal/mol. Partial atomic charges were calculated using the Gasteiger–Huckel method. Surflex-Dock implements an incremental construction search approach as in Hammerhead and also a new fragment assembly methodology for docking [32, 33]. It uses a flexible molecular docking algorithm that combines the empirical scoring function from the Hammerhead with a new molecular similarity method to generate binding poses of ligand fragments in the receptor binding site. Surflex-Dock score is represented in the units of –log (K_d_) to represent the binding affinities of the ligand [32]. The high resolution X-ray crystallographic structure of EGFR was taken from protein data bank (www.rcsb.org) which has binding affinity for the well-known cancer drug, gefitinib (PDB ID: 4WKQ), an EGFR-tyrosine kinase inhibitor. The same binding site was used to dock honokiol. Gefitinib was also docked in to the same binding site for comparison.

### Statistical analysis

The statistical significance of the difference between the values of control and treatment groups was determined by either Student *t* test or simple one-way ANOVA using GraphPad Prism program (San Diego, CA). In each case *P* < 0.05 was considered statistically significant.
